# Gender Affirmation–Related Information-Seeking Behaviors in a Diverse Sample of Transgender and Gender-Diverse Young Adults: Survey Study

**DOI:** 10.2196/45952

**Published:** 2023-08-15

**Authors:** Elizabeth R Boskey, Meg Quint, Rena Xu, Jessica Kremen, Carlos Estrada, Regina Tham, Kaiden Kane, Sari L Reisner

**Affiliations:** 1 Division of Gynecology Boston Children's Hospital Boston, MA United States; 2 Department of Social and Behavioral Sciences Harvard T.H. Chan School of Public Health Boston, MA United States; 3 Department of Surgery Harvard Medical School Boston, MA United States; 4 Department of Endocrinology, Hypertension, and Diabetes Brigham and Women’s Hospital Boston, MA United States; 5 The Fenway Institute Fenway Health Boston, MA United States; 6 Department of Urology Boston Children's Hospital Boston, MA United States; 7 Division of Endocrinology Boston Children's Hospital Boston, MA United States; 8 Department of Pediatrics Harvard Medical School Boston, MA United States; 9 Department of Epidemiology Harvard T.H. Chan School of Public Health Boston, MA United States; 10 Department of Medicine Harvard Medical School Boston, MA United States

**Keywords:** web-based health, information seeking, transgender youth, health communication, survey, gender diverse, youth, United States, support, gender identity, accessibility, transgender community, web-based information

## Abstract

**Background:**

Of the 1.6 million transgender and gender-diverse (TGD) people in the United States, approximately 700,000 are youth aged 13-24 years. Many factors make it difficult for TGD young people to identify resources for support and information related to gender identity and medical transition. These range from lack of knowledge to concerns about personal safety in the setting of increased antitransgender violence and legislative limitations on transgender rights. Web-based resources may be able to address some of the barriers to finding information and support, but youth may have difficulty finding relevant content or have concerns about the quality and content of information they find on the internet.

**Objective:**

We aim to understand ways TGD young adults look for web-based information about gender and health.

**Methods:**

In August 2022, 102 young adults completed a 1-time survey including closed- and open-ended responses. Individuals were recruited through the Prolific platform. Eligibility was restricted to people between the ages of 18-25 years who identified as transgender and were residents of the United States. The initial goal was to recruit 50 White individuals and 50 individuals who identified as Black, indigenous, or people of color. In total, 102 people were eventually enrolled.

**Results:**

Young adults reported looking on the internet for information about a broad range of topics related to both medical- and social-gender affirmation. Most participants preferred to obtain information via personal stories. Participants expressed a strong preference for obtaining information from other trans people.

**Conclusions:**

There is a need for accessible, expert-informed information for TGD youth, particularly more information generated for the transgender community by members of the community.

## Introduction

Of the 1.6 million transgender and gender-diverse (TGD) people in the United States, approximately 700,000 are youth aged 13-24 years [1]. Due to experiences of minority stress, TGD youths are substantially more likely to experience depression, anxiety, and other mental health concerns, including attempted suicide [2-5]. This risk is increased among multiply marginalized Black, indigenous, or people of color (BIPOC) youth—those who experience stigma and discrimination across multiple aspects of their identity, including race and gender identity [6]. Social and medical gender affirmation have been shown to decrease mental health comorbidities [7], as has the presence of supportive adults in a young TGD person’s life [8,9].

Many factors make it difficult for TGD young people to get the information and support they need. These range from lack of knowledge of resources to government actions that are actively hostile to both the existence of TGD youths and their access to medical care. Though social and medical gender affirmation are widely acknowledged to be essential for the health and well-being of TGD youths and young adults [10], as of March 2022, 15 states had restricted or were considering restricting gender-affirming medical care through legislation, which if all passed would deprive 58,000 youths of access to necessary medical care [11]. Other states pursued legislative attacks against social affirmation of or medical care for TGD youths using regulatory barriers imposed by state medical boards or by designating gender affirmation as child abuse [12]. Threats to criminalize gender affirming care may also make it difficult for medical providers to share information about both medical and social affirmation with their young patients due to concerns such as loss of medical licensure or imprisonment. The American Academy of Pediatrics, the American Medical Association, and other major medical associations oppose legal action that restricts access to gender affirming care, based on the risk it poses to the well-being of TGD people [13]. The World Professional Association for Transgender Health Standards of Care Version 8, the current international standards for the providers of transgender health care, underscores the widespread recognition that gender-affirming care is medically necessary for both youths and adults [10].

Outside of legislation restricting access to care, TGD youths experience many other barriers to accessing information about gender and health. Despite broad support by medical providers and organizations for gender-affirming care, many health care providers have not received adequate training in providing care and support for TGD youths [14,15]. Many youths also lack access to consistent support in school and at home [14,15].

TGD youths often turn to web-based sources for social support [9,16], community connection [9,16], information about both social and medical gender affirmation, and other health-related information [17]. The amount of TGD health information available through mainstream video and audio media platforms such as YouTube and TikTok is rapidly rising [18]. On these sites, most of the content is posted by TGD individuals undergoing care [19]. TGD information can be found on several web-based platforms, including text-based general websites such as Tumblr and Reddit, and TGD-specific sites like Transbucket, where users can find crowdsourced materials, such as pre- and postoperative photos for gender confirmation surgeries from various gender-affirming providers. Despite an acknowledgment of the benefits of increased access to web-based sources, both youths and supportive caregivers may have concerns about the quality and content of information they find on the internet. Specifically, youths may be wary of misinformation, the use of outdated language and guidelines, and of engaging with harmful and “disturbing” antitransgender rhetoric or hate speech while searching for information relevant to gender and gender affirming care [9].

The growing legislative and rhetorical attacks on TGD youths and caregivers are often fueled by intentional misinformation about the risks and benefits of both social and medical gender affirmation. As such, there is a clear need for accurate information about gender affirmation that is accessible to TGD youths and their caregivers and provided in spaces that are safe and explicitly welcoming. Most existing studies of the use of web-based spaces by TGD youths and caregivers are limited by a variety of factors, including being focused on the use of a single social media platform [16,19,20], containing locationally bound samples [21], being heavily caregiver focused [9], or focusing on community connection rather than medically related information. This underscores a gap in research exploring information-seeking behaviors among TGD youths who wish to learn about social and medical gender affirmation.

TransHealthGUIDE is a funded National Institutes of Health project (1U01OD033248-01) designed to reduce health disparities and address poor mental health outcomes for TGD youths, especially youths of color, through a multilevel intervention for youths, caregivers, and providers. One intervention component aims to provide youths and caregivers with access to a digital platform containing evidence-based, personalized content related to social and medical gender affirmation, mental health, and community support. While the overall structure of the intervention was informed by expert guidelines from World Professional Association for Transgender Health [10], University of California, San Francisco [22], and the research and clinical expertise of the intervention team, early in-depth key informant interviews of TGD youths, caregivers, and care providers identified a need to better understand (1) the types of information TGD youths are seeking, (2) the places youths currently look for such information, and (3) the format in which youths prefer to obtain information related to gender affirmation in order to inform the final structure and content of the intervention. This exploratory survey was designed to expand on and quantify our understanding of the information needs of TGD youths.

## Methods

### Study Overview

This study was developed as an extension of the key informant interview process for the multilevel TransHealthGUIDE intervention project, a National Institutes of Health–funded U01 multilevel intervention designed to improve access to care and support for gender-diverse youths. The primary youth-facing intervention will be a mobile app to provide resources and support, and a key informant interview process was designed to improve the utility of the final product by making certain it would address participant needs. Initial key informant interview guides were developed through an iterative process of discussion with researchers and staff members who either identified as part of the transgender umbrella or who had substantial clinical or research experience with this population. After the initial round of key informant interviews were completed, there was a recognized need for feedback from larger numbers of diverse young TGD individuals than were accessible for the interviews, which this protocol was designed to elicit. The goal of this survey was to identify critical content areas and styles for the proposed app-based intervention.

### Ethics Approval

After review, this protocol was declared exempt by the Boston Children’s Hospital Institutional Review Board (IRB-P00043127).

### Recruitment

Participants were recruited using the Prolific survey platform, as the site is known to have a large, diverse sample available including many transgender young adults. Recruitment on Prolific allows for the selection of an anonymous, targeted demographic sample within their overall participant panel consisting of an international sample of individuals ages 18 years and older. In this case, our recruitment strategy was to request a sample of young adults between the ages of 18 and 25 years who lived in the United States and identified as transgender according to platform screening questions, which may be updated by users at any time. Two separate samples of 50 individuals who identified as White and 50 individuals who identified as a race other than White were recruited for this protocol to mirror our intended demographics for the overarching project. The platform stops issuing invitations, and deletes unanswered invitations, when the prespecified sample size is reached. Although the study automatically closed after 100 responses were collected, 2 individuals experienced technical difficulties when trying to take the survey and did not complete the protocol before the study slots were full. They anonymously reached out to the study team expressing that they wished to participate and were given the opportunity to do so. As such, a total of 102 individuals completed the survey. Of note, 4 individuals who were classified as non-White by the platform screener self-identified as White on the survey, and they were classified as White in the analysis.

Individuals who were eligible to participate in the study received an invitation using the Prolific platform [23] between August 29, 2022, and August 31, 2022. If after reading the study description they chose to participate, they were directed to the survey (Multimedia Appendix 1), which was housed on the Boston Children’s Hospital Research Electronic Data Capture (REDCap) site [24,25]. After completing the survey, which took on average slightly longer than the estimated 10 minutes (13 minutes, 3 seconds), they were returned to Prolific platform using a completion code. Participants were approved for a US $3 payment within 24 hours of completing the survey. This corresponded to greater than US $13 per hour, which is substantially higher than the required minimum of US $8 per hour required by Prolific. Participants remained fully anonymous.

### Measures

The survey, which included both structured and open-ended response elements, was designed to include the core domains that had been established during the key informant process but achieve greater granularity in areas central to the technical and behavioral design aspects of the project. Items were also included in the survey that specifically addressed areas where key informants had indicated a need for additional data. Prior to launch, the survey was reviewed by a diverse range of staff to assure it was both comprehensive and affirming of identity.

### Analysis

Qualitative data from open-ended questions were analyzed by question or topic to understand specific issues around content development for TGD youths. A conventional content analysis of open-ended survey responses was performed by 2 reviewers (ERB and MQ) in Microsoft Word [26,27]. Reviewers independently analyzed and identified both themes and the number of people who endorsed them. Discrepancies, primarily in phrasing of themes, were resolved through a process of discussion. The number of nonnull respondents for each question is included in the caption of the analysis. Null responses were inclusive of only “no,” “no response,” or “n/a” typed into the field, and these were not counted or analyzed. Quantitative analyses were descriptive as no a priori hypotheses were being tested, given the exploratory nature of this study. Individuals were categorized as BIPOC if they endorsed at least 1 racial or ethnic identity other than White. Data are presented as frequencies and percentages for categorical variables or means and SDs for continuous variables. Descriptive statistics are stratified and presented for BIPOC, White, and all TGD young people in the sample. All analyses were implemented in Stata (version 16.0; StataCorp).

## Results

### Demographics

Characteristics of the study sample are displayed in Table 1. Overall, 102 gender-diverse individuals between the ages of 18 and 25 were recruited into the study. While the goal was to recruit a balanced sample, the platform screening misclassified some individuals who self-identified as White as BIPOC, which led to a slight oversampling of White participants.

**Table 1:** Sample characteristics of transgender and gender-diverse young adults in the United States (N=102).

				BIPOC^a^ participants^b^ (n=46), n (%)		White participants (n=56), n (%)		Total, n (%)
**Gender identity^c^**								
	**Feminine identities**							
		Woman	3 (6)		6 (11)		10 (10)	
		Transgender woman	7 (15)		3 (5)		13 (13)	
		Transfeminine	2 (4)		33 (59)		5 (5)	
	**Masculine identities**							
		Man	7 (15)		9 (16)		16 (16)	
		Transgender man	12 (26)		12 (21)		24 (24)	
		Transmasculine	6 (13)		8 (14)		14 (14)	
	**Nonbinary identities**							
		Nonbinary	25 (54)		33 (59)		58 (57)	
		Agender	2 (4)		7 (12)		9 (9)	
		Bigender	1 (2)		0 (0)		1 (1)	
		Genderqueer	4 (9)		7 (12)		11 (11)	
		Genderfluid	5 (11)		11 (20)		16 (16)	
		Two-spirit	0 (0)		0 (0)		0 (0)	
	**Other identities**							
		Not cisgender or label uncertain	0 (0)		1 (2)		1 (1)	
		Another	1 (2)		4 (7)		5 (5)	
**ASAB^d^**								
	Female			33 (72)		44 (79)		77 (66)
	Male			13 (28)		12 (21)		25 (34)
**Race^c^**								
	American Indian or Alaskan Native			5 (11)		0		5 (5)
	Asian			15 (33)		0		15 (15)
	Black			12 (26)		0		12 (12)
	Hispanic or Latino/a/x			22 (49)		0		22 (22)
	Native Hawaiian or Pacific Islander			2 (4)		0		2 (2)
	White			18 (39)		55 (100)		73 (72)
	Another option or missing			2 (4)		0		4 (4)
**Location where respondent grew up**								
	Midwest			5 (11)		14 (25)		19 (19)
	Northeast			8 (17)		15 (27)		23 (22)
	South			19 (41)		19 (34)		38 (37)
	West			14 (30)		8 (14)		22 (22)
**Caregiver religion^c,e^**								
	N/A^f^			5 (11)		5 (9)		10 (10)
	Agnostic			4 (9)		11 (20)		15 (15)
	Atheist			5 (11)		13 (23)		18 (18)
	Buddhist			5 (11)		0 (0)		5 (5)
	Catholic			22 (48)		18 (32)		40 (39)
	Evangelical			1 (2)		2 (4)		3 (3)
	Jewish			1 (2)		5 (9)		6 (6)
	Mormon			0 (0)		3 (5)		3 (3)
	Mainline Protestant			3 (6)		4 (7)		7 (7)
	Orthodox			6 (13)		10 (18)		16 (16)
	Spiritual or not religious			3 (6)		5 (9)		8 (8)
	Unaffiliated			1 (2)		3 (5)		4 (4)
	Other			6 (9)		5 (9)		11 (11)
**Used source *at least sometimes* to learn about gender identity, gender affirmation, or gender transition as a teenager^c^**								
	Social media			41 (89)		43 (77)		84 (82)
	Websites			36 (78)		39 (70)		75 (73)
	YouTube			31 (67)		33 (59)		64 (62)
	Friends			30 (65)		32 (57)		62 (60)
	Books			9 (20)		14 (25)		23 (22)
	Community organizations			8 (17)		7 (12)		15 (15)
	Health care organizations or providers			5 (11)		10 (18)		15 (15)
	School professionals			4 (9)		3 (5)		7 (7)
PHQ-2^g^ score, mean (SD)				3.0 (2.0)		2.4 (1.9)		2.6 (1.9)

^a^BIPOC: Black, indigenous, or people of color.

^b^Endorses at least 1 racial or ethnic identity other than White.

^c^Adds to greater than 100% because people could say yes to multiple choices.

^d^ASAB: assigned sex at birth.

^e^The following religions were each endorsed by fewer than 3 participants and were added to the “Other” group: Jehovah’s Witness, Muslim, pagan, and Unitarian.

^f^”N/A” was an option on the survey that individuals could select. The meaning was not specified.

^g^PHQ-2: Patient Health Questionnaire-2**.**

Of the BIPOC study population, approximately half identified as Hispanic or Latino/a/x, a third identified as Asian, and a quarter identified as Black. Individuals were allowed to select multiple racial identity options, and more than half (24/46) of individuals in this group selected more than 1 racial identity for their self-description. Geographically, there was a slightly higher proportion of individuals who grew up in the South of the United States compared to the Northeast, Midwest, or West, particularly within the BIPOC study population. There was also substantial religious diversity among the population’s caregivers. The largest proportion of individuals identified as having been raised Catholic (40/102), followed by Orthodox (16/102), atheist (18/102), and agnostic (15/102). The mean Patient Health Questionnaire-2 score of 2.6 suggested a tendency toward mild depression in this population (Table 1).

### Information Sources and Topics

When asked about what information sources they had used to access information about gender and health as a teenager, social media was by far the most commonly used option (84/102). However, more than half of individuals also identified using websites (75/102), YouTube (64/102), and friends (62/102) as sources of information (Table 1). The most common topics participants had searched for were “talking about gender identity” (69/102), “nonmedical ways to express gender” (64/102), “finding support” (63/102), “finding web-based community” (60/102), “finding mental health support” (57/102), “telling people about their gender or coming out” (51/102), “how to transition in various contexts” (49/102), “sexuality and/or sexual health” (49/102), “medical gender care” (49/102), “social transition” (48/102), and “legal transition” (45/102). A substantial fraction (70-94/102) of individuals stated that the information they found for most of those topics was at least moderately useful. However, less than 80% of people stated that information was at least moderately useful for several of the most commonly searched topics, including nonmedical ways to express gender, medical transition, and sexual health (Table 2).

**Table 2:** Transgender and gender-diverse young adults who (1) looked for information at least sometimes on a specific topic as a teenager or (2) indicated information on that topic would have been at least moderately useful for them as teenagers (N=102).

Topic	Looked for information *at least sometimes* as a teenager, n	Indicated information would have been *at least moderately useful* to them as a teenager, n
Talking about gender identity (in general)	69	92
Nonmedical ways to express gender (binding or tucking)	64	79
Finding support for yourself	63	94
Finding a web-based community or meeting other people going through similar things	60	83
Finding mental health support or a therapist	57	86
Telling people about your gender (coming out)	51	82
How to transition in various contexts	49	78
Sexuality and/or sexual health	49	78
Hormones and other medical gender care	49	70
Social transition	48	81
Legal transition	45	74
Surgical gender care	39	60
Talking about gender identity (with family or caregivers)	32	78
Finding support for your family	20	61
Puberty blockers	12	56

When individuals were asked to identify the specific websites they preferred for accessing information about gender and health, the most commonly identified sites were YouTube (64/102), Reddit (62/102), Twitter, (53/102), TikTok (48/102), and Tumblr (44/102). When asked what types of content they liked for learning about gender and health, the most popular styles and formats of content were personal stories (70/102), followed by text (63/102), short videos (1-3 min, 60/102), and infographics (59/102). By far, individuals’ preferred sources for gender-focused content were trans people in general (84/102) and trans friends (83/102). Individuals could select multiple response options (Table 3).

**Table 3:** How and where transgender and gender-diverse young people sampled in the United States prefer to get information “about gender and health?” (N=102).

Questions/responses			Values, n
**Which, if any, of the following websites/social media platforms do you use to get information about gender and health?**			
	YouTube	64	
	Reddit	62	
	Twitter	53	
	TikTok	48	
	Tumblr	44	
	Instagram	36	
	Discord	28	
	Facebook	8	
	Transbucket	7	
	Snapchat	4	
	Other specific sites^a^	3	
**What types of content do you like for learning about gender and health?**			
	Personal stories	70	
	Text	63	
	Infographics	60	
	Videos 1-3 min	59	
	Videos 4-9 min	49	
	Videos ≥10 min	43	
	Comics	41	
	Research papers	29	
	Podcast	25	
	Worksheets	15	
**From what types of people do you like to get information about gender and health?**			
	Trans people in general	84	
	Trans friends	83	
	Medical professionals	52	
	Trans influencers	50	
	Researchers	44	
	Educators	18	
	Media sources	16	
	Caregivers of trans people	14	

^a^Other sites included Animo Transgender Community, Spotify, and Trevorspace.

### Topics That Participants Would Have Wanted Information About as Teenagers

Qualitative data were analyzed by question or topic to understand specific issues around content development for TGD youths. Approximately half the sample (49/102) responded to at least 1 open-ended question, and 35 of 102 (34%) responded to 2 or more. In the first question young adults listed several topics on which they would have liked information when they were growing up. These ranged from general topics, such as understanding gender identity development and evolution, to more specific topics reflecting their individual circumstances and needs. Young adults commented on the need for support in finding TGD-affirming medical care, navigating familial, relational, and cultural contexts, and finding community. There were also requests for information about narrower topics, such as neopronouns and voice training (Textbox 1).

**Content analysis of themes from responses to “Are there other topics you would have liked to have access to information on when you were growing up?” (38 nonnull respondents).**

**Themes (respondents, n)**

- General information
  General information about gender (3)
  “Everything” (2)
  Different types of dysphoria or euphoria (2)
  What it means to be transgender (1)
  That it’s normal for gender identity to change over time (1)
  Gender stereotypes and how to break them (1)
- Medical transition
  Better hormone replacement therapy resources (2)
  Finding trans affirming doctors (1)
  How to get treatment when family is unsupportive (1)
- Family and community
  Finding local community (2)
  More on legal transition (1)
  How to encourage allies to correct names and pronouns (1)
  How to be out to others when not out to family (1)
  How to talk to family in different cultural contexts (1)
  How to navigate relationships with cisgender partners (1)
  Transitioning safely in unaccepting areas(1)
- Specific topic needs
  Gender and religion (2)
  Sexuality and gender (1)
  Sex education (1)

**Illustrative quotes**

All of it. I struggled to find an identity for years because of the amount of trans erasure in my community. Even now I feel strongly that I won’t be accepted unless I pass and pass immaculately. It’s very painful.

Asian transgender woman from Michigan, aged 24 years

I think that hearing from people about their experiences medically transitioning made it easier for me to see if i wanted to go through and start hormones. (Speaking from a ftm pov) i know that it was kind of scary just knowing things would change but not having much anecdotal information. hearing first hand how much things changed, about how long it changed, anything to be wary of, etc. would have been helpful to understand.

White, Latino/x, transmasculine, nonbinary, agender person from Arizona, aged 21 years

Understanding the difference between being self-conscious and being dysphoric; Gender Euphoria.

White, transmasculine, nonbinary, gender-fluid person from South Carolina, aged 24 years

### Topics That Participants Would Want Included in the Project App

When asked about additional topics they thought should be included in the final app, there were multiple requests for information in broad areas, such as “LGBT topics” and comprehensive lists of identities. A number of young people mentioned the need for information that addressed complexities of intersectionality and cross-cultural identity development around gender. Requests were made for specific inclusion of topics relevant to transmasculine and nonbinary youths (Textbox 2).

**Content analysis of themes from responses to “Are there any topics that we haven’t asked about that you think should be addressed in our phone app?” (24 nonnull respondents).**

**Themes (respondents, n)**

- Broad information requests
  Comprehensive list of identities or trans umbrella (3)
  Gender at different ages (2)
  More about social transition (2)
  How to start talking about gender, not just coming out (1)
  General awareness of lesbian, gay, bisexual, transgender, queer topics (1)
  Stories of positive and negative experiences after transition (1)
  “Typical” pubertal development for trans kids (1)
  Gender euphoria (1)
  How dysphoria and euphoria can manifest (1)
  More to life than just being trans (1)
- Race and culture
  Complexities of race and gender or intersectionality (3)
  Cross-cultural histories of gender identity or queer history (2)
  Intercultural language barriers for transition (1)
- Inclusion of specific groups
  More nonbinary inclusion (3)
  Specific info for trans men (1)
- Specific topic needs
  Sexuality and gender (2)
  Neopronouns (1)
  Voice training (1)
  Legal transition in different states (1)
  Finding local social support (1)
  The informed consent model versus gatekeeping (1)
  Continuing health care when you become an adult (1)
  Dating as a queer or trans person (1)
  Navigating relationship with genitals (1)
  Sex education (1)
  Identifying that you are in an abusive situation (1)
  Safety (1)
  Being intersex (1)
  Puberty blocker awareness (1)
  Navigating older parents (1)
- Religious caregivers and trans identity (1)

**Illustrative quotes**

Expand on the complexities between race and gender identity, as well as the ways to address them. Introduce information about the colonialist histories that reinforces binary, cis-only gender identities. There were plenty of cultures that had different views of gender identity before western intervention. It would be powerful to see how various cultures are now learning to overcome colonial impacts on their ideas of gender.

Asian, nonbinary person from Maryland, aged 24 years

How to start the conversation about gender. whether it be a coming-out conversation or not but just how to talk about gender because we don’t do that a society in general.

White, Latino/a/x, nonbinary, genderqueer person from California, aged 20 years

How “typical” social-emotional development is different for young trans folks--talking about “second puberty” for kids who come out post-puberty, and having to start from square one with the whole identity development thing. Also dating as a queer/trans person.

White, nonbinary, gender queer person from New Jersey, aged 25 years

### Specific Challenges Faced by BIPOC Youths

BIPOC young adults endorsed several specific challenges related to race and ethnicity when trying to access information or care. Some of these concerns reflected difficulty communicating with parents about concepts, such as nonbinary gender, that were difficult to discuss in highly gendered languages (eg, Spanish) and cultural contexts. Others involved navigating racial stereotypes or feelings of isolation around their intersecting racial and gender minority identities (Textbox 3).

**Content analysis of themes from BIPOC youth responses to “Are there specific challenges you have faced when getting care or looking for information related to your race and/or ethnicity?” (11 nonnull respondents).**

**Themes (respondents, n)**

- Being accepted by family (2)
- Lack of resources for people from specific racial or ethnic groups (2)
- Finding material in parents’ native language (1)
- Navigating explicitly gendered languages (1)
- Not feeling alone as a racial minority lesbian, gay, bisexual, transgender, queer person (1)
- Booking appointments with doctors (1)
- Feeling awkward around same race or culture providers(1)
- Sexualization of race and gender (1) or bias related to racial stereotypes (1)

**Illustrative Quotes**

Feels super awkward around asian doctors because they remind me of my parents and i feel like they can get really judgy.

Asian, nonbinary person from Maryland, aged 21 years

It was hard to find translations of LGBTQ+ terms in my parents’ native language, much less terms that related to my trans identity. It made it all the more discouraging to even breach the topic with them.

Asian, nonbinary person from Maryland, aged 24 years

### Privacy and Security Concerns

Young adults raised several potential privacy and safety concerns about the proposed project. Some young adults expressed fears about hacking and doxing (publishing someone’s private or identifiable information on the internet with malicious intent), while others were more concerned about family members or parents finding the app on their phone. They were very clear that strong data protection—as well as password protection and a way to rapidly exit the app or switch to an app not related to lesbian, gay, bisexual, transgender, queer (LGBTQ+) topics—would be necessary to minimize risk to participants. Several people also stated that the app should not be explicitly gender-related in appearance to protect users. There were fewer concerns about accessibility, although the need for alt text (descriptive text about an image’s contents) and closed captioning were mentioned (Textbox 4).

**Content analysis of themes from responses to “Do you have any specific privacy or accessibility concerns that you think we should address in the app?” (31 nonnull respondents).**

**Themes (respondents, n)**

- Protect data or have clear policies on data sharing (12)
- Disguise app as not specifically gender related, including notifications (7)
- Add a password (5)
- Have an escape method or stealth mode (4)
- Concerns about parents being able to access app (3)
- Fear of hacking or doxing (3)
- Accessibility in small towns (1)
- Make certain content accessible (alt text or closed captioning) (1)

**Illustrative quote**

I would put in a sort of kill switch to more quickly close or otherwise hide the app if need be, and to provide thorough end-to-end encryption if applicable. I would also enforce a policy if not giving info to authorities if they’re looking for it to enforce anti-trans laws like I know some menstruation/pregnancy apps do.

White, Latino/a/x, transgender man from Florida, aged 24 years

## Discussion

### Principal Findings

TGD young adults endorsed searching for a wide variety of information about gender affirmation, health, and social needs on the internet. However, importantly, their answers also suggested that, as young adults, they have become aware of a number of topics that would have been useful for them to learn about when they were younger, topics that at the time they did not know to look for. This suggests a need for broader education and discussion of gender identity development across society, as the lack of language for a concept can make it difficult to locate information about that concept. While there is no high-quality evidence suggesting that learning about gender diversity leads to increased adoption of gender-diverse identities (ie, the debunked social contagion hypothesis), our clinical experience suggests that many TGD young people struggle to understand how they fit into the world when they are raised in environments that don’t discuss their existence as a possibility [12,28,29].

When looking at the specific topics of interest, the following were all of interest to the TGD young people in this study: hormones, and surgical care, other medical gender-affirming care, mental health support or therapy, and sexuality or sexual health. However, the most searched topics reflected more general questions and concerns about gender identity and nonmedical ways to express it, something that has also been reported in previous studies of web-based behavior [30]. Given the data collected, we cannot ascertain the reason why fewer participants reported searching for information on medical care for gender affirmation. Reluctance to search for information on medical care could result from concerns about privacy or lack of desire for medical affirmation, or both. This finding may also reflect the gender identities of the individuals surveyed, which skewed heavily toward nonbinary identities, a group that may be less likely to be interested in medical gender affirmation as compared to binary transgender youths. The heavy weighting of our survey questions toward medical affirmation reflected the planned intervention being designed by medical providers, but it may also reflect some unconscious assumptions around trans normativity that posit a gender journey inclusive of some degree of medical and surgical affirmation being of interest to the majority of transgender youths, when that is not necessarily the case [31-33]. Indeed, that concern was raised by several respondents, and a de-emphasis on binary and medicalized genders has been incorporated into the app design.

More than 20% of study participants stated they had trouble finding information about nonmedical gender affirmation, which may include changing one’s name and pronouns; or using hairstyling, clothing, makeup, padding, or body-modifying garments to create an appearance that more accurately reflects a person’s internal sense of self. Nonmedical gender affirmation may also involve binding or tucking to conceal highly gendered areas of the body that are associated with discomfort or dysphoria. Both improper binding and tucking have the potential to lead to injury, ranging from skin breakdown [34] and infection to testicular torsion [35]. Accurate information about the benefits of social gender affirmation may be useful to help young people enact them in as safe a way as possible and to advocate for their needs with family, school, and other environments.

TGD youths expressed a significant desire for resources that facilitate connection to other members of their community. This likely reflects both the importance of social engagement for well-being and the challenges in identifying support experienced by members of marginalized or multiply marginalized groups, particularly during the COVID-19 pandemic [36]. An interest in community connection may also underlie participants’ strong preference for learning information about gender and health from other TGD people. Given the preponderance of politically motivated misinformation campaigns targeted at TGD youths, a preference for trans-produced content may also reflect conscious or subconscious attempts to screen for content that is less likely to be transphobic or otherwise hostile—TGD young people scanning for safety. While not yet studied in TGD communities, there is also growing literature about the advantages of being treated by others with similar identities for people from racially minoritized groups [37-39].

Participants’ interest in content from TGD people aligns with a strong preference among respondents for learning specifically from the personal stories of other TGD people, a theme that was also articulated in key informant interviews performed with youths and caregivers as part of the overarching project (data not yet published) and also reported by other investigators doing similar work [40]. Historically, digital storytelling has served as an effective learning tool for patients, caregivers, and providers and a mechanism for community outreach in other areas [41]. This suggests tremendous potential for its use in a TGD health intervention. While youths using internet sources for health information used Google to initiate searches and visited medical websites more often than social media platforms, TGD youths primarily used social networking websites for gender-related and health information [28]. This difference may be due to lack of accessible, age-appropriate, and reliable gender-affirming information on health-specific websites [42].

As part of this formative evaluation, we were interested in determining whether TGD young people had strong preferences for styles of information as well as source when seeking information related to gender and health. Previous research has suggested a preference for interactive content and visual content (videos, images, and animation) among adolescents seeking general health information on the internet, but it was unclear whether these preferences would convey to TGD young people [43]. In this study, the most popular platform for information-seeking was YouTube (video-based platform or application—not inherently social or interactive). Reddit (text- and photo-based discussion forum) and Twitter (multimedia platform—highly interactive) were also very highly endorsed; both are text-based platforms. Surprisingly, although TikTok (short videos—strong social content) was brought up frequently in individual key informant interviews and was the second most frequently used social media platform among US teenagers in 2021, it was not one of the top information sources in this survey [44]. This may reflect how survey questions were framed: in this survey, participants were asked to reflect on the sources they previously used, which may have been prior to TikTok’s creation. The preference for text over videos of any length was also reflected in the types of content for which young people expressed a preference, suggesting the importance of varied content types to support young people’s information-seeking behaviors.

Another important aspect of intervention development raised by participants was the need to protect the data and privacy of individuals interacting with any TGD-focused resources and media. For youths whose TGD identity is unknown to their caregivers, disclosure of this information may put them at risk of abuse or housing loss. TGD youths with unsupportive caregivers have become unhoused as well as experienced both physical and emotional abuse [45,46]. In addition, in the current regulatory environment, supportive caregivers may be at risk of having custodial rights revoked, or undergoing investigation by child protective organizations [12]. Given safety concerns, and in line with suggestions from participants, plans for our proposed interventions now include ensuring information is available in a form that is not clearly visibly branded as trans to a casual observer and creating a quick escape option similar to those seen on websites and apps designed as resources for individuals experiencing abuse [47].

This study has several limitations. Individuals must be aged 18 years or older to join the Prolific platform, so we were unable to survey those aged under 18 years. This is potentially important, as youths aged under 18 years may have different preferences due to a different life stage and our planned educational intervention has a target audience that includes individuals aged 13-25 years. In addition, there is a risk of selection bias; the sample of TGD young people using Prolific is almost certainly not representative of the population of TGD young people as a whole, likely being at a minimum more computer literate and having needed to hear about the platform to enroll. Participants were asked to recall informational sources, unmet needs, and topics from their early teens, which may have introduced recall bias; the accuracy of self-reporting on past information-seeking behaviors is unknown. Preferred information sources have also likely shifted in the years since the participants reached the age of majority, as young people’s social media preferences change substantially over time. Strengths of this exploratory study include enrolling a purposive sample of TGD young adults from across the United States comprised of 50% BIPOC youths, gathering exploratory quantitative and qualitative data informed by findings from key informant interviews, and centering on the experiences of young TGD people.

### Public Health Implications

The findings of this study underscore the need for accessible, expert-informed information for TGD youths, particularly more information generated for the TGD community, by the TGD community. TGD-identified and TGD health and research experts have suggested a “nothing about us without us” framework [48,49], which can be adapted for use in research with TGD young people. TGD youths are looking, with mixed success, for information on a wide range of topics, from general overviews of gender identity to more specific questions about gender affirmation, building community, and sexual health. There is a need for intentional interaction between community, researchers, and medical professionals to generate and codevelop trustworthy resources on these topics. Findings suggest that involvement of and presentation by trans people is a key element of establishing trust with TGD youths. Further, there is a need for information to be presented using multiple methods (ie, video, text, and audio) to meet the varied preferences of youths. Finally, youths identify web-based safety as a critical area for content providers, and individuals working in this field should recognize and address the fears that TGD youths have in both web-based and in-person spaces.

## Appendix

Multimedia Appendix 1 Survey.

## Abbreviations

BIPOC: Black, indigenous, or people of color
LGBTQ+: lesbian, gay, bisexual, transgender, queer, and other sexual and gender minority identities
REDCap: Research Electronic Data Capture
TGD: transgender and gender-diverse
