# Supplementary material for: Gender Affirmation–Related Information-Seeking Behaviors in a Diverse Sample of Transgender and Gender-Diverse Young Adults: Survey Study
Source: JMIR Form Res. 2023 Aug 15;7:e45952. doi: 10.2196/45952 (PMC10466148; doi:10.2196/45952)
Supplement: Multimedia Appendix 1 [file formative_v7i1e45952_app1.pdf]

How young adults find out information about gender and health

We are reaching out to inform you about an opportunity to participate in a research study at Boston Children's Hospital. This study is being conducted by Elizabeth Boskey, PhD and the TransHealthGUIDE team. Individuals are eligible to participate if they identify as transgender, live in the United States, and are between the ages of 18-25 years old.

This survey will be used to develop online tools to help transgender, non-binary, and gender expansive youth and their caregivers and families feel supported, find medical and community resources, and get accurate information about transgender care.

For part of the project, we're also working directly with providers to increase the number of people who feel comfortable providing or referring to gender affirming care.

If you want to learn more, you can check out our webpage at transhealthguide.info

If you choose to participate you will complete the following survey that has been approved by the Institutional Review Board at Boston Children's Hospital. It should take less than 10 minutes. No identifiable information will be collected about you and there is no direct benefit for your completion of the survey.

Risks are minimal and primarily reflect discomfort with answering the questions. You may skip any question that makes you feel uncomfortable.

Thank you for taking the time to read the above information. By beginning the survey you consent to take our survey and have your answers collected and analyzed. You will be paid $3 once your survey is approved.

By completing our survey/questionnaire, you are giving your consent to participate in the research study as described above. Thank you for your interest.

If you have any questions, please contact Elizabeth Boskey - elizabeth.boskey@childrens.harvard.edu

NOTE: Individuals who fail both attention checks will have their responses manually reviewed by investigators before they are accepted, and such responses may be rejected without payment.

Please enter your Prolific ID

____

What is your current age (in years)

____
(Whole number years only)

What is your gender identity?
(select all that apply)

- ☐ Man
- ☐ Transgender man
- ☐ Transmasculine
- ☐ Woman
- ☐ Transgender woman
- ☐ Transfeminine
- ☐ Non-binary
- ☐ Agender
- ☐ Genderqueer
- ☐ Genderfluid
- ☐ Bi-gender
- ☐ Two-spirit
- ☐ Not-cisgender but don't have a term
- ☐ Another gender identity: ______

What is your sex assigned at birth?

- ○ Male
- ○ Female

What racial and ethnic categories best describe you? (check all that apply)	☐ White<br>☐ Black or African American<br>☐ Hispanic or Latina/o/x<br>☐ Asian<br>☐ American Indian or Alaska Native<br>☐ Native Hawaiian or Other Pacific Islander<br>☐ Another race/ethnicity: ______<br>☐ Prefer not to answer
At what age did you first realize that you had a different gender identity from what was expected for your sex assigned at birth?	◯ Under 5 years old<br>◯ 6-8 years old<br>◯ 9-12 years old<br>◯ 13-15 years old<br>◯ 16-18 years old<br>◯ After 18 years old

What state do you currently live in most of the time?	◯ Alabama<br>◯ Alaska<br>◯ Arizona<br>◯ Arkansas<br>◯ California<br>◯ Colorado<br>◯ Connecticut<br>◯ Delaware<br>◯ Florida<br>◯ Georgia<br>◯ Hawaii<br>◯ Idaho<br>◯ Illinois<br>◯ Indiana<br>◯ Iowa<br>◯ Kansas<br>◯ Kentucky<br>◯ Louisiana<br>◯ Maine<br>◯ Maryland<br>◯ Massachusetts<br>◯ Michigan<br>◯ Minnesota<br>◯ Mississippi<br>◯ Missouri<br>◯ Montana<br>◯ Nebraska<br>◯ Nevada<br>◯ New Hampshire<br>◯ New Jersey<br>◯ New Mexico<br>◯ New York<br>◯ North Carolina<br>◯ North Dakota<br>◯ Ohio<br>◯ Oklahoma<br>◯ Oregon<br>◯ Pennsylvania<br>◯ Rhode Island<br>◯ South Carolina<br>◯ South Dakota<br>◯ Tennessee<br>◯ Texas<br>◯ Utah<br>◯ Vermont<br>◯ Virginia<br>◯ Washington<br>◯ West Virginia<br>◯ Wisconsin<br>◯ Wyoming
Is the area you currently live in best described as:	◯ Urban<br>◯ Suburban<br>◯ Rural<br>◯ I don't know

What state did you live in most of the time prior to age 18?

- ◯ Alabama
- ◯ Alaska
- ◯ Arizona
- ◯ Arkansas
- ◯ California
- ◯ Colorado
- ◯ Connecticut
- ◯ Delaware
- ◯ Florida
- ◯ Georgia
- ◯ Hawaii
- ◯ Idaho
- ◯ Illinois
- ◯ Indiana
- ◯ Iowa
- ◯ Kansas
- ◯ Kentucky
- ◯ Louisiana
- ◯ Maine
- ◯ Maryland
- ◯ Massachusetts
- ◯ Michigan
- ◯ Minnesota
- ◯ Mississippi
- ◯ Missouri
- ◯ Montana
- ◯ Nebraska
- ◯ Nevada
- ◯ New Hampshire
- ◯ New Jersey
- ◯ New Mexico
- ◯ New York
- ◯ North Carolina
- ◯ North Dakota
- ◯ Ohio
- ◯ Oklahoma
- ◯ Oregon
- ◯ Pennsylvania
- ◯ Rhode Island
- ◯ South Carolina
- ◯ South Dakota
- ◯ Tennessee
- ◯ Texas
- ◯ Utah
- ◯ Vermont
- ◯ Virginia
- ◯ Washington
- ◯ West Virginia
- ◯ Wisconsin
- ◯ Wyoming

When you were growing up, which of the following types of people lived with you and/or were responsible for taking care of you? (check all that apply)

- ☐ Mom(s)
- ☐ Dad(s)
- ☐ Other Parent(s)
- ☐ Grandparent(s)
- ☐ Other relatives
- ☐ Foster family
- ☐ Social Worker/State Worker
- ☐ Other: ______

Do you identify as religious or as a member of a particular religious group?<br>(select all that apply)	☐ N/A<br>☐ Agnostic<br>☐ Athiest<br>☐ Buddhist<br>☐ Catholic<br>☐ Evangelical Protestant<br>☐ Hindu<br>☐ Jehovah's Witness<br>☐ Jewish<br>☐ Latter-day Saint/Mormon<br>☐ Mainline Protestant<br>☐ Muslim<br>☐ Orthodox Christian<br>☐ Pagan<br>☐ Sikh<br>☐ Unitarian/Univeralist<br>☐ Spiritual but not religious<br>☐ Unaffiliated<br>☐ Other ______
Do your immediate family, or the people who raised you, identify as religious or as members of a particular religious group?<br>(select all that apply)	☐ N/A<br>☐ Agnostic<br>☐ Athiest<br>☐ Buddhist<br>☐ Catholic<br>☐ Evangelical Protestant<br>☐ Hindu<br>☐ Jehovah's Witness<br>☐ Jewish<br>☐ Latter-day Saint/Mormon<br>☐ Mainline Protestant<br>☐ Muslim<br>☐ Orthodox Christian<br>☐ Pagan<br>☐ Sikh<br>☐ Unitarian/Univeralist<br>☐ Spiritual but not religious<br>☐ Unaffiliated<br>☐ Other ______
How old were you when you first started to think about your gender or question your gender?	◯ 5 years old or younger<br>◯ 6-8 years old<br>◯ 9-12 years old<br>◯ 13-15 years old<br>◯ 16-18 years old<br>◯ Older than 18 years old
How old were you when your parents or other caregivers found out that you were transgender?	◯ 5 years old or younger<br>◯ 6-8 years old<br>◯ 9-12 years old<br>◯ 13-15 years old<br>◯ 16-18 years old<br>◯ Older than 18 years old<br>◯ They don't know
How old were you when you read this question that is designed to check if you are paying attention?<br><br>If you are paying attention, please select "Older than 18 years old"	☐ 5 years old or younger<br>☐ 6-8 years old<br>☐ 9-12 years old<br>☐ 13-15 years old<br>☐ 16-18 years old<br>☐ Older than 18 years old

Which of the following is true about how your parents or caregivers learned that you were transgender? (check all that apply)	☐ I told (all of) them<br>☐ I told (some of) them<br>☐ A friend of mine told them (or told someone who told them)<br>☐ A doctor, teacher, or other professional who I had told, told them<br>☐ They don't know<br>☐ Other
Do you currently have health insurance? (check all that apply)	☐ No<br>☐ Yes, Medicare<br>☐ Yes, Medicaid<br>☐ Yes, Other Public Insurance<br>☐ Yes, Private Insurance (for example, from employer or school)<br>(Note: State medicaid programs have various different names, if you're not certain, select "other public insurance")
When you were growing up, what type of insurance did you/your family have? (check all that apply)	☐ No<br>☐ Yes, Medicare<br>☐ Yes, Medicaid<br>☐ Yes, Other Public Insurance<br>☐ Yes, Private Insurance (for example, from employer or school)<br>☐ I don't know<br>(Note: State medicaid programs have various different names, if you're not certain, select "other public insurance")
Do you think your race and/or ethnicity affected how hard it was for you to come out and/or understand your gender identity?<br><br>If so, did it make it:	○ Much harder<br>○ Slightly harder<br>○ Neither harder nor easier<br>○ Somewhat easier<br>○ Much easier<br>○ It's complicated<br>○ N/A
Do you think you or your family's religious beliefs affected how hard it was to come out and/or understand your gender identity?<br><br>If so, did it make it:	○ Much harder<br>○ Slightly harder<br>○ Neither harder nor easier<br>○ Somewhat easier<br>○ Much easier<br>○ It's complicated<br>○ N/A
Do you think the place where you grew up affected how hard it was for you to come out and/or understand your gender identity?<br><br>If so, did it make it:	○ Much harder<br>○ Slightly harder<br>○ Neither harder nor easier<br>○ Somewhat easier<br>○ Much easier<br>○ It's complicated<br>○ N/A

Over the last 2 weeks, how often have you been bothered by the following problems?	Not at all	Several days	More than half the days	Nearly every day
Little interest or pleasure in doing things	○	○	○	○
Feeling down, depressed or hopeless	○	○	○	○

The next group of questions will ask about how you looked for information about gender identity or care options when you were a teenager.

When you were a teenager, how often did you learn about gender identity, gender affirmation, or gender transition from the following sources:

	Never	Occasionally	Sometimes	Often	Very Often
Social Media	◯	◯	◯	◯	◯
YouTube	◯	◯	◯	◯	◯
Websites	◯	◯	◯	◯	◯
Books	◯	◯	◯	◯	◯
Friends	◯	◯	◯	◯	◯
Community organizations	◯	◯	◯	◯	◯
Clinics, hospitals, other healthcare	◯	◯	◯	◯	◯
Teachers, or other school professionals	◯	◯	◯	◯	◯
Another place: ______	◯	◯	◯	◯	◯

When you were a teenager, how often were you looking for information about the following topics:

	Never	Occasionally	Sometimes	Often	Very Often
Telling people about your gender ("coming out")	◯	◯	◯	◯	◯
Talking about gender identity (in general)	◯	◯	◯	◯	◯
Talking about gender identity (with family/caregivers)	◯	◯	◯	◯	◯
Finding support for yourself	◯	◯	◯	◯	◯
Finding support for your family/caregivers	◯	◯	◯	◯	◯
Finding online community / Meeting other people going through similar things as I was	◯	◯	◯	◯	◯
Social transition	◯	◯	◯	◯	◯
How to transition in various contexts	◯	◯	◯	◯	◯
Legal transition, such as changing your name or ID card	◯	◯	◯	◯	◯
Non-medical ways to express gender (e.g. binding/tucking/etc.)	◯	◯	◯	◯	◯
Puberty blockers	◯	◯	◯	◯	◯
Hormones & other medical gender care	◯	◯	◯	◯	◯
Surgical gender care	◯	◯	◯	◯	◯
Finding mental health support or a therapist	◯	◯	◯	◯	◯
Sexuality and/or sexual health as a trans person	◯	◯	◯	◯	◯
Another topic: ______	◯	◯	◯	◯	◯

When you were a teenager, how useful would it have been to have reliable information about the following topics:

	Not at all	Slightly	Moderately	Very	Extremely
Telling people about your gender (i.e. "coming out")	○	○	○	○	○
Talking about gender identity (in general)	○	○	○	○	○
Talking about gender identity (with family/caregivers)	○	○	○	○	○
Finding support for yourself	○	○	○	○	○
Finding support for your family/caregivers	○	○	○	○	○
Finding online community / Meeting other people going through similar things as I was	○	○	○	○	○
Social transition	○	○	○	○	○
Non medical ways to express gender (i.e. binding/tucking/etc)	○	○	○	○	○
Legal transition, such as changing your name or ID card	○	○	○	○	○
How to transition in various contexts	○	○	○	○	○
Puberty blockers	○	○	○	○	○
Hormones and other medical gender care	○	○	○	○	○
Surgical gender care	○	○	○	○	○
Finding mental health support or a therapist	○	○	○	○	○
Sexuality & sexual health as a trans person	○	○	○	○	○
Another topic: ______	○	○	○	○	○

The next set of questions are about your relationship with your parents or caregivers when you were a teenager.

How much trouble did your parents or caregivers have with each of the following:

	None	A little	A moderate amount	A lot	Not applicable/They don't know I'm trans
Understanding your gender identity	○	○	○	○	○
Accepting your gender identity	○	○	○	○	○
Using correct pronouns and/or name	○	○	○	○	○
Providing emotional support	○	○	○	○	○
Providing financial support (in general)	○	○	○	○	○
Talking to my school about my gender identity/name/pronouns	○	○	○	○	○
Talking to other family members (cousins, grandparents, etc.) about my gender identity/name/pronouns	○	○	○	○	○
Finding doctors and other resources for you	○	○	○	○	○
Helping to pay for gender-related expenses or care	○	○	○	○	○
Addressing their religious beliefs	○	○	○	○	○
If you are paying attention, please select "A moderate amount"	○	○	○	○	○

Did you and/or your family use any of the following materials to learn about gender and/or help with your relationship?

	Neither	I used	My parents/caregivers used	Both
Online educational material	◯	◯	◯	◯
Online stories/social media	◯	◯	◯	◯
Books (fiction)	◯	◯	◯	◯
Books (non-fiction)	◯	◯	◯	◯
Educational material from school	◯	◯	◯	◯
Educational material from a doctor or medical office	◯	◯	◯	◯
Educational materials from a therapist or other mental health professional	◯	◯	◯	◯
Support groups	◯	◯	◯	◯
Professional therapy	◯	◯	◯	◯
Conversation guides	◯	◯	◯	◯
Talking with clergy/religious consultation	◯	◯	◯	◯
Something else: ______	◯	◯	◯	◯

How useful were online educational material for helping you with your relationship with your caregivers?

◯ Not at all useful
◯ Slightly Useful
◯ Somewhat Useful
◯ Very Useful

How useful were online stories/social media for helping you with your relationship with your caregivers?

◯ Not at all useful
◯ Slightly Useful
◯ Somewhat Useful
◯ Very Useful

How useful were books(fiction) for helping you with your relationship with your caregivers?

◯ Not at all useful
◯ Slightly Useful
◯ Somewhat Useful
◯ Very Useful

How useful were books(nonfiction) for helping you with your relationship with your caregivers?

◯ Not at all useful
◯ Slightly Useful
◯ Somewhat Useful
◯ Very Useful

How useful were online stories/social media for helping you with your relationship with your caregivers?

◯ Not at all useful
◯ Slightly Useful
◯ Somewhat Useful
◯ Very Useful

How useful was educational material from school for helping you with your relationship with your caregivers?

◯ Not at all useful
◯ Slightly Useful
◯ Somewhat Useful
◯ Very Useful

How useful was educational material from a doctor or medical office for helping you with your relationship with your caregivers?	◯ Not at all useful<br>◯ Slightly Useful<br>◯ Somewhat Useful<br>◯ Very Useful
How useful was educational material from a therapist or mental health professional for helping you with your relationship with your caregivers?	◯ Not at all useful<br>◯ Slightly Useful<br>◯ Somewhat Useful<br>◯ Very Useful
How useful were support groups for helping you with your relationship with your caregivers?	◯ Not at all useful<br>◯ Slightly Useful<br>◯ Somewhat Useful<br>◯ Very Useful
How useful was professional therapy for helping you with your relationship with your caregivers?	◯ Not at all useful<br>◯ Slightly Useful<br>◯ Somewhat Useful<br>◯ Very Useful
How useful were conversation guides for helping you with your relationship with your caregivers?	◯ Not at all useful<br>◯ Slightly Useful<br>◯ Somewhat Useful<br>◯ Very Useful
How useful was talking with clergy or other religious professionals for helping you with your relationship with your caregivers?	◯ Not at all useful<br>◯ Slightly Useful<br>◯ Somewhat Useful<br>◯ Very Useful

This next set of questions will ask about your interest in, and experience with, gender affirming care as a teenager

As a teenager, how interested were you in getting any of the following:

	Not at all	Slightly	Moderately	Very	Extremely	N/A
Puberty blockers (medications to stop you from going through puberty)	◯	◯	◯	◯	◯	◯
Hormones to help your body match your gender	◯	◯	◯	◯	◯	◯
Surgery to help your body match your gender	◯	◯	◯	◯	◯	◯
Therapy - General	◯	◯	◯	◯	◯	◯
Therapy - Related to your gender	◯	◯	◯	◯	◯	◯
Non-medical gender support items (clothes, binders, gaffs, etc.)	◯	◯	◯	◯	◯	◯

When you tried to get healthcare related to your gender, how often were each of the following a problem?

	Never	Occasionally	Sometimes	Often	Very Often
Knowing that a type of care was possible	◯	◯	◯	◯	◯
Finding a doctor within a reasonable distance	◯	◯	◯	◯	◯
Long wait times to get an appointment	◯	◯	◯	◯	◯
Doctor refusing to provide treatment	◯	◯	◯	◯	◯
Insurance didn't cover care	◯	◯	◯	◯	◯
Cost of treatment	◯	◯	◯	◯	◯
Getting to treatment (because of distance, taking time off, transportation, or for other reasons)	◯	◯	◯	◯	◯
Caregiver/parent didn't agree or support you in getting gender-related healthcare	◯	◯	◯	◯	◯
Being made uncomfortable by a doctor/therapist	◯	◯	◯	◯	◯
Fear of doctor/therapist disclosing gender identity to family	◯	◯	◯	◯	◯
Another topic: ______	◯	◯	◯	◯	◯

If you had the opportunity to participate in live-chats or other group sessions with a gender care doctor, how useful would you find (or have found) the following topics?

	Not at all	Slightly	Moderately	Very	Extremely
Using medication to stop puberty	○	○	○	○	○
Medical gender care	○	○	○	○	○
Surgical gender care	○	○	○	○	○
Fertility (having children)	○	○	○	○	○
Transitioning in various environments	○	○	○	○	○
How to get gender care	○	○	○	○	○
Talking to family or friends about gender identity	○	○	○	○	○
Finding local care	○	○	○	○	○
Sexuality/Sexual health	○	○	○	○	○

If you had the opportunity to get additional information about gender care and could choose three topics, which three would you choose? (Rank top 3 choices)

	First	Second	Third
Using medication to stop puberty	○	○	○
Medical gender care	○	○	○
Surgical gender care	○	○	○
Fertility (having children)	○	○	○
Transitioning in various environments	○	○	○
How to get gender care	○	○	○
Talking to family or friends about gender identity	○	○	○
Finding local care	○	○	○
Other: ______	○	○	○

Are there other topics you would like to have had access to information on when you were growing up?

____

Are there specific challenges you have faced when getting care or looking for information related to your race and/or ethnicity?

____

The next set of questions will ask you about how you like to get information. For the purposes of these questions, assuming you're using an app on your phone.

Which, if any, of the following websites/social media platforms do you use to get information about gender and health?
(check all that apply)

- ☐ Tiktok
- ☐ YouTube
- ☐ Instagram
- ☐ Snapchat
- ☐ Facebook
- ☐ Discord
- ☐ Tumblr
- ☐ Reddit
- ☐ Twitter
- ☐ Transbucket
- ☐ Other specific site ______

What type of content do you like for learning about gender and health?
(check all that apply)

- ☐ Videos 1-3 minutes
- ☐ Videos - 4-8 minutes
- ☐ Videos - 10 minutes or longer
- ☐ Infographics (i.e. fun graphics with information)
- ☐ Podcasts
- ☐ Text
- ☐ Worksheets (fillable forms)
- ☐ Comics
- ☐ Personal Stories
- ☐ Research papers
- ☐ Wiki -style article
- ☐ Frequently Asked Questions
- ☐ Live question & answer session
- ☐ Blog posts
- ☐ Internet Forum (i.e. Reddit, Facebook groups)

From what types of people do you like to get information about gender and health?

- ☐ Trans friends
- ☐ Trans influencers
- ☐ Trans people in general
- ☐ Medical professionals
- ☐ Teachers & other educational professionals
- ☐ Parents and caregivers of trans people
- ☐ Researchers
- ☐ Media sources

How do you determine if information about gender and health are reliable?

____

The next set of questions are entirely optional. You can hit submit and return to Prolific, if you don't have additional information to add.

Are there any topics that we haven't asked about that you think should be addressed in our phone app?

____

Do you have any specific privacy or accessibility concerns that you think we should address in the app?

____

Are there any topics that you think are ONLY appropriate for a trans youth OR a caregiver? If so, what are they?

____

Is there anything else you would like to share that you think is relevant to our project design, or are there topics you would like to expand on from earlier in the survey?

____
